# Noise risk assessment practices of four South African manufacturing and utilities companies

**DOI:** 10.4102/sajcd.v70i1.996

**Published:** 2023-11-28

**Authors:** Oscar Rikhotso, Thabiso J. Morodi, Daniel M. Masekameni

**Affiliations:** 1 Department of Environmental Health, Faculty of Science, Tshwane University of Technology, Pretoria, South Africa; 2 Department of Occupational Health, Faculty of Health Sciences, University of the Witwatersrand, Johannesburg, South Africa

**Keywords:** monitoring, occupational hygienists, review, risk management, stakeholder participation

## Abstract

**Background:**

The South African Noise Induced Hearing Loss (NIHL) Regulations, mandates employers to conduct a noise risk assessment, which records specific variables for determining the status of exposure and the need for implementation of control measures.

**Objectives:**

The study evaluated company noise risk assessment practices for alignment with legal requirements and specific risk assessment guidelines.

**Method:**

Convenience sampling was used to select the four manufacturing and utilities companies that participated in the study. The participating companies submitted latest noise risk assessment records for evaluation through the READ approach.

**Results:**

The noise risk assessment records of three of the four companies omitted the recording of factors such as the reasonable deterioration in or failure of control measures, adequate control and formalisation of hearing conservation programmes (HCPs). When evaluated against the South African National Standard 31000 Risk Assessment guidelines, the risk assessment processes of the respective companies were lacking in addressing aspects related to establishing communication and consultation, evaluation, adapting, continually improving, leadership and commitment, and integration.

**Conclusion:**

The recorded information on the noise risk assessment reports from the four participating companies were incomplete, negatively affecting subsequent HCP management processes and decision-making. Future studies should investigate other aspects such as the implementation status of recommended noise controls as well as their effectiveness as recorded in the noise risk assessment records.

**Contribution:**

This study provided firsthand insights of company noise risk assessment practices, specifically identifying functional and technical areas requiring improvement to enhance current efforts directed towards the minimisation of NIHL within HCPs. The study highlighted that the current practices on recording noise risk assessment information remain incomplete, adversely diminishing the impact of the assessment as an important decision-making tool. The identified technical issues specifically, when addressed, will increase trust on the decisions derived from noise risk assessments.

## Introduction

Occupational health programmes, including hearing conservation programmes (HCPs), offer employers a risk management tool for managing and minimising the impacts of exposure (Khattab, 1987) and are instituted based on the findings of a risk assessment (South African National Standard, 2018, 2021). Various workplace regulations in South Africa including the Noise Induced Hearing Loss (NIHL) Regulations, mandate employers to conduct risk assessments encompassing all occupational health hazards (South Africa, 2002, 2003, 2020, 2021). Companies that voluntarily adopt complementary occupational health and safety (OHS) systems such as the South African National Standard (SANS) 45001 also require carrying out risk assessments through the identification and recognition of hazards and risks as an important step of the risk assessment process (South African National Standard, 2018). Successful OHS management relies on information derived from the risk assessment as a decision-making tool (Health and Safety Executive [HSE], 2003), highlighting its importance in risk management.

## Purpose and need for conducting risk assessments

A risk assessment is vital within the overall risk management process and includes aspects such as communication and consultation, context establishment, risk analysis, risk evaluation, risk treatment, monitoring and review (South African National Standard, 2009, 2010, 2019). A risk assessment is intentionally conducted to determine the acceptability of workplace risks (HSE, 2003), to determine and select available preventive control measures for risk reduction (South African National Standard, 2010). In determining and selecting risk reduction measures, the risk assessment information is assessed to inform decision-making which also considers available technical resources, the prevailing social, economic and political values (Cohrssen & Covello, 1989). The risk assessment information also forms the basis for risk management, a contemporary central issue in OHS policies (Rantanen, 1981; South African National Standard, 2019). Within the risk-based decision-making framework, risk management options are identified in the initial risk assessment phase where problem formulation and scoping are defined. This therefore implies that a risk assessment is only rendered to be an evaluation mechanism for the risk reduction strategies as opposed to being an end product (Robinson & Levy, 2011).

### Risk assessment process

The entire risk assessment process is in itself a time-consuming activity (Eds. Pitblado & Turney, 1996), which is administratively demanding and burdensome to companies (HSE, 2006). Procedurally, the information about risks and hazards is gathered using methods which include field surveys, pre-job assessments and facility assessments (Center for Chemical Process Safety, 2010). Practically, risk assessments are conducted on hypothetical persons in fixed workstations close to hazards based on the premise that risk control decisions should not rely on a person being exposed to hazards first. However, individual risk assessments can also be conducted as the need arises. The hypothetical person technique has merit as it permits risk being underestimated by distinguishing the appropriateness of a generic assessment of the risks. For compliance purposes, however, checks are done to ascertain whether actual workers have similar occupational risk profile to that of a hypothetical person assumed in the assessment (HSE, 2001).

### Risk assessment and legal compliance

From an OHS legal perspective, worldwide, risk assessments are conducted by employers as a quest towards demonstrating compliance with relevant legislation (South Africa, 1993, 1996; United Kingdom, 1974, 1999). Labour inspectorates have strategically assigned the responsibility of conducting risk assessments with employers, in recognition of internal limitations of its expertise in the face of technological advancement on processes and applied technology used in the workplace (Russ, 2010). Within the South African context, the resources for carrying out the risk assessment are provided by the affected companies (South African National Standard, 2010). Employers delegate the function of conducting risk assessments to professionals deemed to have relevant technical knowledge of the process as well as knowledge on associated legal requirements. Companies conducting risk assessments gain deeper understanding of risks and are enabled to identify contributory factors to risk management system weaknesses (South African National Standard, 2010). Additionally, workers in such companies have an increased awareness of workplace hazards not previously identified while business benefits deriving therefrom includes bringing more business opportunities, contribution to the companies’ safety record and avoiding negative media publicity arising from workplace accidents (Center for Chemical Process Safety, 2010; O’Hara, Dickley & Weyman, 2005) and workload reduction (Center for Chemical Process Safety, 2010). A noise risk assessment is conducted on a prescribed biennial frequency for existing plant installations, whereas an immediate review is conducted following changes in process work methods and type of activities carried out, as well as availability of new technology for noise control which affects prevailing risk levels (South Africa, 2003; South African National Standard, 2021).

### Noise risk assessments, noise induced hearing loss and hearing conservation programmes

Noise induced hearing loss prevalence from various industries in South Africa, indicates that hazard elimination has seemingly been impossible. According to Rikhotso et al. (2022b), publicly available occupational disease statistics from South African general industry indicates that a total of 19 084 NIHL cases were compensated between 2001 and 2020. This indicates the importance of proper and responsive noise risk management, which is grounded on risk assessments and exposure control (Center for Chemical Process Safety, 2010). Information derived from the noise risk assessment process informs the design of policies, exposure control strategies and their implementation (Cohrssen & Covello, 1989). This thus implies, from a South African regulatory perspective that, risk assessments are an important cog in the establishment of occupational health programmes (De Jager et al., 2014). A noise risk assessment within the context of HCPs incorporates elements such as noise monitoring, audiometry, noise training programme and noise reduction (South African National Standard, 2021). The minimum information to be recorded in noise risk assessments in South Africa is prescribed in Regulation 6 of the NIHL Regulations (South Africa, 2003), while the South African National Standard (2021) outlines additional variables to be recorded. How noise risk assessment information is collected and utilised is at the discretion of regulated industry. This implies methodological variances on how South African companies are assessing noise and ensuing risk management processes.

## Aim and objectives

This study enrolled company noise risk assessment records, with the aim of evaluating the alignment of recorded information with prescriptions outlined in the NIHL Regulations and recommended guidelines outlined in the South African National Standard (SANS) 31000.

## Research methods and design

### Company identification and selection, and data collection process (noise risk assessment report submission)

The participating companies were conveniently selected, informed by a risk-based criteria of confirmed historic NIHL incidence, determined through the evaluation of sustainability reports. The final selected companies were identified and selected using a longitudinal study of 20 manufacturing and utilities companies, the method of which has been previously reported by Rikhotso et al. (2023; 2022a). Company A is from the utilities sector, whereas Companies B (petroleum refining), C (radioisotope manufacturing) and D were from the manufacturing sector. Companies A and C had 11 and 6 operational units, respectively. Both Companies B and D had two operational units, each. The included companies submitted recent electronic noise risk assessment records for each operational unit for evaluation, which were securely stored in a password-protected folder by the primary investigator (O.R.) upon receipt.

### Report evaluation criteria

The submitted noise risk assessment records were evaluated based on a criteria derived from the NIHL Regulations (subregulations 6, 9 and 10) (South Africa, 2003), the SANS 10083 (South African National Standard, 2021) and SANS 31000 (South African National Standard, 2019) requirements. Subregulations 6, 9 and 10 of the NIHL Regulations as well as the SANS 10083 guidelines prescribe the recording of the sources of noise exposure, adverse effects of exposure to excessive noise levels, extent of worker exposure, the nature of work process and reasonable deterioration in failure of control measures during noise risk assessments (South Africa, 2003; South African National Standard, 2021). The recording of these factors informs the risk analysis outcomes of the risk assessment process. Furthermore, the submitted noise risk assessment records were also evaluated against the SANS 31000 Risk Assessment guidelines including the recording of framework definition, evaluation criteria, design, implementation, evaluation and improvement (South African National Standard, 2019).

### Data analysis

The READ (Ready materials, Extract data, Analyse data, Distil) approach (Dalglish et al., 2020) to document analysis was used in the evaluation of the submitted noise risk assessment records. The READ approach to document analysis includes successive steps of readying the materials, data extraction, data analysis and distillation of the findings (Dalglish et al., 2020). The extracted qualitative data were input onto Microsoft word-generated tables as appears on Table 1 to Table 4.

**TABLE 1:** Personnel conducting noise risk assessments.

Company and facility	Occupational hygiene assistant	Occupational hygiene technologist	Occupational hygienist	Occupational health nurse or practitioner	Safety officer	Risk officer
**Company A**						
Facility A1	-	✓	-	-	-	-
Facility A2	✓	-	-	-	-	-
Facility A3	-	✓	-	-	-	-
Facility A4	-	✓	-	-	-	-
Facility A5	✓	-	✓	-	-	-
Facility A6	-	✓	✓	-	-	-
Facility A7	-	✓	✓	-	-	-
Facility A8	-	✓	-	✓	-	-
Facility A9	-	-	✓	-	-	-
Facility A10	-	✓	-	-	-	-
Facility A11	-	-	✓†	-	-	-
**Company B**						
Facility B1	-	✓	✓	-	✓	
Facility B2	-	✓	✓	-	✓	
**Company C**						
Facility C1	-	✓	-	-	-	-
Facility C2	-	-	✓	-	-	-
Facility C3	-	✓	-	-	-	-
Facility C4	-	-	✓	-	-	-
Facility C5	-	✓	-	-	-	-
Facility C6	-	✓	-	-	-	-
**Company D**						
Facility D1	-	-	-	-	-	✓
Facility D2	-	-	-	-	-	✓

✓, Confirmed.

† , Consulting occupational hygienist.

**TABLE 2:** Recorded stakeholder involvement and participation during noise risk assessments.

Company and facility	Employer (representative)	Health and safety representative	Process representatives	Occupational health nurse or practitioner	Safety officer	Other stakeholders
**Company A**						
Facility A1	✗	✗	✗	✗	✗	✗
Facility A2	✗	✗	✗	✗	✗	✓†
Facility A3	✗	✗	✗	✗	✗	✗
Facility A4	✗	✗	✗	✗	✗	✗
Facility A5	✗	✗	✗	✗	✗	✗
Facility A6	✗	✗	✗	✗	✗	✗
Facility A7	✗	✗	✗	✗	✗	✗
Facility A8	✗	✗	✗	✓	✗	✗
Facility A9	✓†	✓†	✓†	✓†	✓†	✓†
Facility A10	✗	✗	✗	✗	✗	✗
Facility A11	✗	✗	✗	✗	✗	✓‡
**Company B**						
Facility B1	✓	✗	✓	✗	✓	✓
Facility B2	✓	✗	✓	✗	✓	✓
**Company C**						
Facility C1	✓	✗	✓	✗	✓	✓
Facility C2	✓	✓	✓	✗	✓	✓
Facility C3	✓	✓	✓	✗	✓	✓
Facility C4	✓	✓	✗	✗	✗	✗
Facility C5	✓	✓	✓	✗	✓	✓
Facility C6	✓	✓	✗	✗	✗	✗
**Company D**						
Facility D1	✗	✗	✗	✗	✗	✗
Facility D2	✗	✗	✗	✗	✗	✗

✗, Absent; ✓, Confirmed.

† , Job categories of stakeholder unspecified.

‡ , Safety health environment department personnel (specific roles unclear).

**TABLE 3a:** Recording of noise risk assessment information at Company A facilities.

Evaluation criteria	Facility										
	A1	A2	A3	A4	A5	A6	A7	A8	A9	A10	A11
**Noise-induced hearing loss regulations requirements**											
Factors taken into account during risk assessment											
Noise sources to which workers are exposed	✗†	✗	✗	✗	✗	✓	✗	✗	✗	✗	✓
Adverse health effects that excessive noise may cause	✓	✓	✓	✓	✓	✓	✓	✓	✓	✓	✓
Extent to which workers may be exposed	✓	✓	✗	✓	✓	✓	✓	✗	✓	✓	✓
Nature of work processes	✓	✓	✓	✗	✗	✓	✓	✓	✗	✗	✓
Any reasonable deterioration in or failure of any control measures	✗	✗	✗	✗	✗	✗	✗	✗	✗	✗	✗
Exposure adequately controlled as per risk assessment outcome: Reason why noise is > noise rating limit identified Controls other than HPDs considered Specific technical HPDs for existing noise used	✗	✗	✗	✗	✗	✓‡	✗	✗	✗	✗	✓‡
**SANS 10083 guidelines**											
Hearing conservation programme	✗§	✓	✗§	✗§	✗§	✗§	✗§	✗§	✓	✗§	✓
Assessment of noise	✓	✓	✓	✓	✓	✓	✓	✓	✓	✗	✓
Demarcation of noise zones	✓	✓	✓	✗	✗	✗	✓	✗	✓	✗	✓
Assessment and reassessment of measurement area	✓	✓	✓	✓	✓	✓	✓	✓	✓	✗	✓
Reduction of noise	✗	✗	✗	✗	✗	✓	✗	✗	✓	✗	✓
Wearing of hearing protectors	✓	✓	✓	✗	✓	✓	✓	✓	✓	✓	✓
Audiometric testing programme	✓	✓	✓	✗	✓	✓	✓	✓	✓	✓	✓
**SANS 31000 risk management guidelines**											
Framework											
Leadership and commitment	✗	✗	✗	✗	✗	✗	✗	✗	✗	✗	✗
Integration	✗	✗	✗	✗	✗	✗	✗	✗	✗	✗	✗
**Design**											
Articulating risk management commitment											
Assigning organisational roles, authorities, responsibilities and accountabilities	✓	✓	✓	✓	✓	✓	✓	✓	✓	✓	✓
Allocating resources	✓	✓	✓	✓	✓	✓	✓	✓	✓	✓	✓
Establishing communication and consultation	✗	✗	✗	✗	✗	✗	✗	✗	✗	✗	✗
**Implementation**	✓	✓	✓	✓	✓	✓	✓	✓	✓	✓	✓
**Evaluat ion**	✗	✗	✗	✗	✗	✗	✗	✗	✗	✗	✗
**Improvement**											
Adapting	✗	✗	✗	✗	✗	✗	✗	✗	✗	✗	✗
Continually improving	✗	✗	✗	✗	✗	✗	✗	✗	✗	✗	✗

HPD, hearing protection device.

✓, Recorded; ✗, Absent.

† , Unclear description of noise sources (generic description).

‡ , Attenuation capabilities of available HPDs considered in assessment.

§ , Not directly mentioned.

**TABLE 3b:** Recording of noise risk assessment information at Companies B, C & D facilities.

Evaluation criteria	Company B (Facility)		Company C (Facility)						Company D (Facility)	
	B1	B2	C1	C2	C3	C4	C5	C6	D1	D2
**Noise-induced hearing loss regulations requirements**										
Factors taken into account during risk assessment										
Noise sources to which workers are exposed	✓	✓	✓	✓	NA	✓	NA	✓	✗	✗
Adverse health effects that excessive noise may cause	✓	✓	✓	✓	NA	✓	NA	✓	✗	✗
Extent to which workers may be exposed	✓	✓	✓	✓	NA	✓	NA	✓	✗	✗
Nature of work processes	✓	✓	✓	✓	NA	✓	NA	✓	✗	✗
Any reasonable deterioration in or failure of any control measures	✗	✗	✗	✗	NA	✗	NA	✗	✗	✗
Exposure adequately controlled as per risk assessment outcome: Reason why noise > noise rating limit identified Controls other than HPDs considered Specific technical HPDs for existing noise used	✗	✗	✓†	✗	NA	✓†	NA	✗	✗	✗
**SANS 10083 guidelines**										
Hearing conservation programme	✓	✓	✗	✗	✗	✗	NA	✗	✗	✗
Assessment of noise	✓	✓	✓	✓	NA	✓	NA	✓	✗	✗
Demarcation of noise zones	✓	✓	✗	✗	NA	✓	NA	✓	✗	✗
Assessment and reassessment of measurement area	✓	✓	✓	✓	NA	✓	NA	✓	✗	✗
Reduction of noise	✓	✓	✗	✗	NA	✗	NA	✗	✗	✗
Wearing of hearing protectors	✓	✓	✓	✓	NA	✓	NA	✓	✗	✗
Audiometric testing programme	✓	✓	✓	✓	✓	✓	✓	✓	✗	✗
**SANS 31000 risk management guidelines**										
Framework										
Leadership and commitment	✓	✓	✓	✓	✓	✓	NA	✓	✗	✗
Integration	✓	✓	✓	✓	✓	✓	NA	✓	✗	✗
**Design**										
Articulating risk management commitment										
Assigning organisational roles, authorities, responsibilities and accountabilities	✓	✓	✓	✓	✓	✓	NA	✓	✗	✗
Allocating resources	✓	✓	✓	✓	✓	✓	NA	✓	✗	✗
Establishing communication and consultation	✓	✓	✓	✓	✓	✓	NA	✓	✗	✗
**Implementation**	✓	✓	✓	✓	✓	✓	NA	✓	✗	✗
**Evaluation**	✗	✗	✗	✗	✗	✗	✗	✗	✗	✗
**Improvement**										
Adapting	✗	✗	✗	✗	✗	✗	✗	✗	✗	✗
Continually improving	✗	✗	✗	✗	✗	✗	✗	✗	✗	✗

HPD, hearing protection device; NA, not applicable (noise sources eliminated and current noise level < 85 dBA rating limit).

✓, Recorded; ✗, Absent.

† , Attenuation capabilities of available HPDs considered in assessment.

**TABLE 4:** Risk assessment review and monitoring.

Company and facility	Risk assessment review (current and previous review dates)		Recording of changes from previous assessment and/or review	Monitoring of controls
	Current	Previous		
**Company A**				
Facility A1	✓	✗	✗	✗
Facility A2	✓	✗	✗	✗
Facility A3	✓	✗	✗	✗
Facility A4	✓	✗	✗	✗
Facility A5	✓	✗	✗	✗
Facility A6	✓	✗	✗	✗
Facility A7	✓	✗	✗	✗
Facility A8	✗	✗	✗	✗
Facility A9	✗	✗	✗	✗
Facility A10	✓	✗	✗	✗
Facility A11	✓	✗	✗	✗
**Company B**				
Facility B1	✓	✗	✗	✗
Facility B2	✓	✗	✗	✗
**Company C**				
Facility C1	✓	✓	✗	✗
Facility C2	✓	✗	✗	✗
Facility C3	✓	✗	✗	✗
Facility C4	✓	✗	✗	✗
Facility C5	✓	✗	✗	✗
Facility C6	✓	✗	✗	✗
**Company D**				
Facility D1	✓	✗	✗	✓
Facility D2	✓	✗	✗	✓

✓, Recorded; ✗, Absent.

### Ethical considerations

The study received ethical clearance from the Tshwane University of Technology (TUT) Faculty Committee on Research Ethics-Science: FCRE 2020/10/015 (FCPS 02) (SCI). The primary investigator (O.R.) signed disclosure agreements with the participating companies, as applicable.

## Results

The confirmation and availability of the noise risk assessment records from the four companies were in response to employer obligations for securing legal compliance and to satisfy internal voluntary requirements. The reporting format of the submitted records diverged between full Microsoft Word, PDF, Microsoft Excel spreadsheets and checklists. The type and format of the noise risk assessment record affects the thoroughness and quality of information contained in such documents.

### Personnel conducting noise risk assessments

Employers from Companies A, B and C delegated the duty of conducting noise risk assessments to occupational hygiene professionals who had varying Southern African Institute for Occupational Hygiene (SAIOH) certification levels ranging from occupational hygiene assistants, occupational hygiene technologist to occupational hygienists (Table 1). These occupational hygiene professionals were resident specialists stationed at the various facilities of the respective companies with one noted case of a consulting (external) occupational hygienist used at Facility A11, Company A. At Company D, however, a risk officer was delegated as the professional conducting noise risk assessments.

### Stakeholder participation during noise risk assessments

The NIHL Regulations prescribe that the assessment be conducted with involvement and participation of various stakeholders. Stakeholders with recorded involvement and participation in the respective company assessments (Table 2) included the employer (employer representative), health and safety representative, process representative, occupational health nurse or practitioner, safety officer and other unspecified stakeholders. The involvement of multiple stakeholders was a common practice at Companies B and C compared to Companies A and D. Occupational health practitioner and safety officer involvement in conducting noise risk assessments was noted at Facility A8, Company A and all facilities at Company B, respectively.

### Recording of noise assessment information

The recorded information in the noise risk assessment records varied between the respective participating companies (Company A [Table 3a], Companies B, C and D [Table 3b]). At Company A, only two of the 11 (*n* = 2, 18%) facilities recorded the noise sources. None of the companies recorded the reasonable deterioration in or failure of control measures. Furthermore, only two of 11 (*n* = 2, 18%) at Company A, two of six (*n* = 2, 33%) at Company C, and none for Companies B and C recorded the factors for determining the adequate control of exposure status. At Company D, none of the noise risk assessment variables outlined by the NIHL Regulations were recorded, a limitation of the use of a checklist as a risk assessment tool.

In addition to the NIHL Regulations-prescribed variables to be recorded in noise risk assessments, the SANS 10083 code of practice also recommends the recording of additional information in the assessment. The recording of the HCP implementation status was 3 of 11 (*n* = 3, 27%) for Company A, whereas none was recorded for Companies C and D. Noise zone demarcation, an administrative control, was recorded in 6 of 11 (*n* = 6, 54.5%) and 2 of 6 (*n* = 2, 33%) at Company A and Company B, respectively. Furthermore, only 3 of 11 (*n* = 3, 27%) facilities at Company A recorded historic noise reduction efforts undertaken by the respective facilities, whereas none of Companies B, C and D recorded the same information, as applicable. None of Company D noise risk assessment records recorded the variables specified in SANS 10083 code of practice.

The noise risk assessment frameworks of all participating companies did not demonstrate tangible evidence of conformance to the SANS 31000 clauses regarding leadership and commitment, integration, establishing communication and consultation, evaluation, adaptation and continual improvement, in general. This is expected against a national gap without a prescribed and adopted standard for conducting risk assessments by regulated industry.

### Review and monitoring

Once completed, the risk assessment should be reviewed, and the effectiveness of implemented control measures be continuously monitored. Companies A, B and C (Table 4) omitted the recording of changes in the assessment and the monitoring of previously instituted controls. The checklist assessment tool used by Company D allowed for the monitoring of previously instituted exposure controls as well as for the recording of changes in the assessment. In general, all participating companies omitted to record the timeline of previous assessment dates for the measurement of statutory reassessment frequency.

## Discussion

At the time of writing, there remained limited publicly available scientific literature on the subject matter and studies of a similar study design to enable result comparisons.

### Industry risk assessment status

The reviewed records attest that participating companies were conducting noise risk assessments for regulatory compliance purposes (HSE, 2006; Russ, 2010), and for satisfying internal voluntary standards. The noise risk assessment records were also indicative that companies had prior knowledge of the existence of and the potential threat posed by prevailing occupational health hazards (John et al., 2014; Kates, 1977), especially occupational noise, inherent of machinery and equipment operated in industrial processes (John et al., 2014; Reinhold et al., 2009). The noise risk assessments also allowed the participating companies to identify at-risk employees to the prevailing hazards and risks (HSE, 1995), and gathered information required to determine the probability of risk occurrence and its subsequent consequence (Ostrom & Wilhemsen, 2012). Furthermore, the identified risks also enabled the participating companies to use internal knowledge to control the hazards or seek external assistance if hazards are beyond the scope of its experience and knowledge (Eds. Pitblado & Turney, 1996). However, the noise risk assessments would have been advantageous to the participating companies if conducted during plant design, to assure that hazard control knowledge has been applied, which informs the content of ensuing safe work procedures used for daily operations (Eds. Pitblado & Turney, 1996). The information gathered during the noise risk assessment process should be systematically and proactively used to respond to these identified risks (South African National Standard, 2018).

### Risk assessors and perceptions of risk

Risk assessors are expected to have thorough understanding and in-depth technical knowledge of a process being assessed to achieve meaningful results (Center for Chemical Process Safety, 2010; Ostrom & Wilhemsen, 2012). Pasman and Rogers (2018) suggest that risk assessors should also have sufficient knowledge about uncertainties related to risk assessment methodologies employed during the assessment, ways of handling them and additional verification methods for validating the risk assessments, all performed to increase trust in its outcomes. The enrolled noise risk assessments were conducted by occupational hygiene professionals as well as a risk officer in one instance (Company D). In other instances, the noise risk assessments were conducted jointly among occupational hygiene professionals, occupational health practitioners and safety officers specifically. As a quality enhancing measure, noise risk assessments should also be peer reviewed before use in any ensuing decision-making process (Ostrom & Wilhemsen, 2012), a practice observable at Companies B and C; certain operational facilities at Company A. Personnel conducting risk assessments matter because of the inherent subjectivity of the process itself. In this regard, three ideologies observed during risk assessments are that some risk assessors are gravitated towards a paradigm that hazards are always greater compared with risks assessed, while others believe that hazards are always less than risks assessed. The third ideology is that prominent hazards differ from those risks, which have been assessed (Kates, 1977). This results in the risk analysis phase of the risk assessment process being mired in disagreement of opinions, biases in risk perception and judgement. The thoroughness of gathered information, assumptions and omissions, however, determines the extent of such professional disagreement of opinions (South African National Standard, 2019). The disagreements arise as individuals perceive and interpret hazards differently based on personal experiences and the nature of hazards. The HSE (2003) advises that in instances where companies engage external consultants to carry out a risk assessment, practice observed at Company A (Facility A11), the process should be properly managed to maintain its participatory nature and to avoid a situation, which can erode the trust of the affected workers on the process. An overreliance on external consultants for conducting risk assessments should be cautiously approached as it can also lead to unrealistic or inappropriate risk conclusions (HSE, 2003).

### Stakeholder participation in noise risk assessments

The multidisciplinary team required for conducting a noise risk assessment (Table 3) should comprise the health and safety representative, occupational hygienist, occupational medical practitioners, among others. The role of the occupational hygienist, occupational medical practitioners and associated professions in noise risk assessments is to facilitate the assessment, identify hazards and risks and provide technical input on available risk reduction measures (Ostrom & Wilhemsen, 2012). The participatory approach in conducting noise risk assessments ensures that the combined views about hazards and risks from different professions are considered, which can result in an increase in risk management efficiency. An additional benefit of risk assessments conducted in a participatory approach is that it equips workers in gaining greater understanding of their roles compared with on the-job training (Eds. Pitblado & Turney, 1996), while also empowering them to take ownership of their health and safety associated with work activities (Center for Chemical Process Safety, 2010). This action is necessary because of the chronic and delayed onset of occupational health impacts (Carson & Henenberg, 1989). Stakeholder participation is even more important for employers, as employers with low awareness levels to the risk assessment process tend to only focus at the process during the start and completion of the risk and hazard identification portion within the broader risk management framework. For this reason, proposals arguing for a change in risk assessment to risk management to encompass the ongoing or continuous nature of the process have credence (HSE, 2006). Stakeholder participation in risk assessment processes should, however, be approached with caution as there is often a tendency to overstate risk levels of hazards (Tziaferi et al., 2011).

### Recording of noise risk assessment information

The noise risk assessment determines and records sources of hazards, exposure levels where available, routes of exposure, exposure duration and frequency, and identification of the most exposed job categories (South Africa, 2003; South African National Standard, 2021; U.S. Environmental Protection Agency [EPA], 2011) (Table 3a and Table 3b). In South African workplaces, the recording of these legislated variables has an impact on the risk analysis phase of the assessment as they have a lessening effect of lowering the probability, severity, exposure or a combination of two or more of the latter options (Jensen, 2012). An HSE (2006) evaluation study on risk assessments found that risk assessments were not complete as they omitted certain operational areas and certain groups of employees in their assessments as well as not completing all risk assessment steps. Furthermore, the HSE evaluation study also found that the practice of risk assessment varies in complexity between companies, with inter- and intra-company variances influenced by geographical locations of operations resulting in a situation whereby hazard identification across an enterprise is inconsistently performed (HSE, 2006). To highlight variances in the risk and hazard identification phases of the risk assessment process, some companies do not have evidence in their risk assessment showing noise is identified as a significant health hazard (Laird et al., 2010). Risk assessment records with incomplete information imply that company risk management might be incorrect: a possible contributory factor in NIHL incidence.

Fragmentation in the risk assessment process, which can be avoided by adopting guidelines such as the SANS 31000, has implications that impact the decision-making process of deciding on implementing required control measures. The risk assessment processes of the participating companies in this study were misaligned with certain selected clauses of SANS 31000 (South African National Standard, 2019), indicative of gaps in the risk management process such as omission of the establishment of the evaluation criteria, the design, implementation, evaluation and improvement guidelines. An absent national directive in South Africa is a contributory factor for the observed gaps in company risk assessment processes.

## Review and monitoring

Following the completion of the entire risk assessment process, practical steps to manage identified health risks should be taken, followed by an evaluation to assess the effectiveness of the effected measures, instruction and training (HSE, 1995), a practice not observed among the participating companies (Table 4). Legally, changes in the risk ranking outcomes recorded following a risk assessment outcomes would trigger a reassessment (HSE, 1995; South Africa, 2003). Apart from changes induced as part of risk control measures, noise risk assessments should be continuously reviewed to track the status of previously identified hazards and to prioritise preventive control measures (Tjoe-Nij et al., 2018), which none of the participating companies did. Thus, to record and measure gains in the risk reduction efforts, currently identified risks should be assessed without consideration of implemented risk reduction measures and be recorded as baseline risk assessment (Jensen, 2012), a practice not observable in either of the participating companies.

## Conclusion

Noise risk assessments were a confirmed practice at the participating companies and were conducted by employer-delegated professionals inclusive of occupational hygienists, occupational health nurse or practitioner as well as a risk officer. Stakeholder involvement and participation during noise risk assessments was recorded extensively at Companies B and C, an indicator of internal communication and consultation in the risk management processes. Legally, health and safety representative and health and safety committee involvement and participation in noise risk assessment is an enforceable legal requirement which was absent in some facilities. Factors such as mention of any reasonable deterioration in or failure of control measures, adequate control and formalisation of HCP, recorded as part of risk assessment variables in the records were generally omitted in the noise risk assessments, adjudging the process as incomplete. The recording of information relating to these factors affects the risk analysis phase. The enrolled risk assessment records were also adjudged as being improperly aligned, in general, to the risk assessment guidelines specified in the SANS 31000 relating to elements such as establishing communication and consultation, evaluation, adapting, continually improving, leadership and commitment, and integration. Additionally, the review and monitoring of the assessed noise risk assessment aspects remained non-existent across all participating companies. Conclusively, the noise risk assessment processes at the four participating companies had technical shortcomings, which influences subsequent HCP management process. In general, an improvement in the noise risk assessment processes among the participating companies is recommended, in order to minimise the pervasive NIHL.

Future studies should investigate the implementation stages and status of recommended noise controls and their effectiveness as outlined in noise risk assessment records. Furthermore, future studies should also investigate the roles of both the health and safety representatives, and health and safety committees in following up on recommended or proposed corrective actions for noise control.

## References

[CIT0001] Carson, W.G., & Henenberg, C. (1989). Social justice at the workplace: The political economy of occupational health and safety laws. *Social Justice*, 16(3), 124–140. Retrieved from http://www.jstor.org/stable/29766488

[CIT0002] Center for Chemical Process Safety. (2010). *A practical approach to hazard identification for operations and maintenance workers*. Center for Chemical Process Safety.

[CIT0003] Cohrssen, J.J., & Covello, V.T. (1989). *Risk analysis: A guide to principles and methods for analysing health and environmental risks*. The Council on Environmental Quality. Executive Office of the President.

[CIT0004] Dalglish, S.L., Khalid, H., & McMahon, S.A. (2020). Document analysis in health policy research: The READ approach. *Health Policy and Planning*, 35(10), 1424–1431. 10.1093/heapol/czaa064 PMC7886435 33175972

[CIT0005] De Jager, N., Beukes, S., & Nolte, A.G.W. (2014). Measuring compliance of conducting occupational health risk assessment in the occupational health nurse’s practice. *HealthSA Gesondheid*, 19(1), 1–11. 10.4102/hsag.v19i1.647

[CIT0006] Health and Safety Executive (HSE). (1995). *Health risk management: A practical guide for managers in SME’s*. Health and Safety Executive.

[CIT0007] Health and Safety Executive (HSE). (2001). *Reducing risks, protecting people. HSE’s decision-making process*. Retrieved from https://www.hse.gov.uk/research/rrhtm/rr279.htm

[CIT0008] Health and Safety Executive (HSE). (2003). *Good practice and pitfalls in risk assessment*. Retrieved from http://www.hse.gov.uk/rsearch/rrpdf/rr151.pdf

[CIT0009] Health and Safety Executive (HSE). (2006). *An evaluation of the five steps of risk assessment*. Health and Safety Executive.

[CIT0010] Jensen, R.C. (2012). *Risk-reduction methods for occupational safety and health*. John Wiley & Sons, Inc.

[CIT0011] John, G.W., Grynevych, A., Welch, D., McBride, D., & Thorne, P.R. (2014). Noise exposure of workers and the use of hearing protection equipment in New Zealand. *Archives of Environmental and Occupational Health*, 69(2), 69–80. 10.1080/19338244.2012.732122 24205958

[CIT0012] Kates, R.W. (1977). Assessing the assessors: The art and ideology of risk assessment. *Ambio*, 6(5), 247–252. Retrieved from https://www.jstor.org/stable/4312292

[CIT0013] Khattab, T.M. (1987). 18 – Occupational health programmes. In A.W. Gardner (Ed.), *Current approaches to occupational health* (pp. 290–296). Butterworth-Heinemann.

[CIT0014] Laird, I., McBride, D., Legg, S., Dickinson, P., McLaren, S., & Gardner, D. (2010). Effective strategies in the prevention of noise induced hearing loss. *New Zealand Acoustics*, 24(3), 4–14. 10.1136/injuryprev-2012-040580a.40

[CIT0015] O’Hara, R., Dickley, N., & Weyman, A. (2005). Good practice in assessing workplace risks by small and medium-sized enterprises. *Risk Management*, 7(1), 31–41. 10.1057/palgrave.rm.8240203

[CIT0016] Ostrom, L.T., & Wilhemsen, C.A. (2012). *Risk assessment. Tools, techniques, and their applications*. A John Wiley & Sons, Inc., Publication.

[CIT0017] Pasman, H., & Rogers, W. (2018). How trustworthy are risk assessment results, and what can be done about the uncertainties they are plaqued with?. *Journal of Loss Prevention in the Process Industries*, 55, 162–177. 10.1016/j.jlp.2018.06.004

[CIT0018] Pitblado, R., & Turney, R. (Eds.). (1996). *Risk assessment in the process industries*. Institution of Chemical Engineers.

[CIT0019] Rantanen, J. (1981). Risk assessment and the setting of priorities in occupational health and safety. *Scandinavian Journal of Work, Environment & Health*, 7(suppl. 4), 84–90. Retrieved from https://www.jstor.org/stable/40964853 7330635

[CIT0020] Reinhold, K., Jarvis, M., & Tint, P. (2009). Risk observatory – A tool for improving safety and health at the workplace. *International Journal of Occupational Safety and Ergonomics*, 15(1), 101–112. 10.1080/10803548.2009.11076792 19272244

[CIT0021] Rikhotso, O., Morodi, T.J., & Masekameni, D.M. (2022a). The extent of occupational health hazard impact on workers: Documentary evidence from national occupational disease statistics and selected South African companies’ voluntary corporate social responsibility disclosures. *Sustainability*, 14(17), 10464. 10.3390/su141710464

[CIT0022] Rikhotso, O., Morodi, T.J., & Masekameni, D.M. (2022b). Occupational health and safety statistics as an indicator of worker physical health in South African industry. *International Journal of Environmental Research and Public Health*, 19(3), 1690. 10.3390/ijerph19031690 35162712 PMC8835012

[CIT0023] Rikhotso, O., Morodi, T.J., & Masekameni, D.M. (2023). An evaluation of occupational health risk assessment methodologies from South African enterprises: Noise risk assessment field study. *Occupational Health Southern Africa*, 29(2), 65–74.

[CIT0024] Robinson, L.A., & Levy, J.I. (2011). The [r]evolving relationship between risk assessment and risk management. *Risk Analysis*, 31(9), 1334–1344. 10.1111/j.1539-6924.2011.01653.x 21740453

[CIT0025] Russ, K. (2010). Risk assessment in the UK health and safety system: Theory and practice. *Safety and Health at Work*, 1(1), 11–18. 10.5491/SHAW.2010.1.1.11 22953158 PMC3430933

[CIT0026] South Africa. (1993). *Occupational Health and Safety Act and Regulations (85 of 1993)*. Retrieved from https://www.gov.za/sites/default/files/gcis_document/201409/act85of1993.pdf

[CIT0027] South Africa. (1996). *Mine Health and Safety Act*. Retrieved from http://www.dmr.gov.za/legislation/summary/30-mine-health-and-safety/530-mhs-act-29-of1996.html

[CIT0028] South Africa. (2001). *Regulations for Hazardous Biological Agents*. Retrieved from https://www.gov.za/sites/default/files/gcis_document/201409/229560.pdf

[CIT0029] South Africa. (2002). *Lead Regulations*. Retrieved from https://www.gov.za/sites/default/files/gcis_document/201409/231750.pdf

[CIT0030] South Africa. (2003). *Noise-induced Hearing Loss Regulations (GNR.307)*. Retrieved from https://www.gov.za/sites/default/files/gcis_document/201409/224990.pdf

[CIT0031] South Africa. (2020). *Asbestos Abatement Regulations*. Retrieved from https://www.gov.za/sites/default/files/gcis_document/202011/43893rg11196gon1196.pdf

[CIT0032] South Africa. (2021). *Regulations for Hazardous Chemical Agents*. Retrieved from https://www.gove.za/sites/default/files/gcis_document/202103/44348rg11263gon280.pdf

[CIT0033] South African National Standard. (2009). Recommended practice. Risk management – Vocabulary. *ARP 070:2009*. SABS Standards Division. pp. 1–15.

[CIT0034] South African National Standard. (2010). Risk management – Risk assessment techniques. *SANS 31010:2010*. SABS Standards Division. pp. 6–118.

[CIT0035] South African National Standard. (2018). Occupational health and safety management systems – Requirements with guidance for use. *SANS 45001:2018*. Standards South Africa. pp. 1–39.

[CIT0036] South African National Standard. (2019). Risk management – Guidelines. *SANS 31000:2019*. South African Bureau of Standards. pp. 1–15.

[CIT0037] South African National Standard. (2021). The measurement and assessment of occupational noise for hearing conservation purposes. *South African National Standard 10083*. Standards South Africa. pp. 5–52.

[CIT0038] Tjoe-Nij, E., Rochin, C., Berne, N., & Sassi, A. (2018). Chemical risk assessment screening tool of a global chemical company. *Safety and Health at Work*, 9(1), 84–94. 10.1016/j.shaw.2017.06.012 30363081 PMC6111129

[CIT0039] Tziaferi, S.G., Sourtzi, P., Kalokairinou, A., Sgourou, E., & Koumoulas, E. (2011). Risk assessment of physical hazards in Greek hospitals combining staff’s perception, experts’ evaluation and objective measurements. *Safety and Health at Work*, 2(3), 260–272. 10.5491/SHAW.2011.2.3.260 22953210 PMC3430906

[CIT0040] United Kingdom. (1974). *Health and Safety at Work Act 1974*. Retrieved from http://www.legislation.gov.uk/ukpga/1974/37/contents

[CIT0041] United Kingdom. (1999). *The management of health and safety at work regulations 1999*. Retrieved from https://www.legislation.gov.uk/uksi/1999/3242/contents/made

[CIT0042] U.S. Environmental Protection Agency (EPA). (2011). *Expsoure factors handbook*. Center for Environmental Assessment. U.S. Environmental Protection Agency.

